# *N*-Cyclo­hexyl-2-(5-fluoro-1*H*-indol-3-yl)-2-oxoacetamide

**DOI:** 10.1107/S1600536811024299

**Published:** 2011-06-30

**Authors:** Dan-Li Tian, Gang Luo, Hong Chen, Xiao-Wei Tang, Yong-Feng Liu

**Affiliations:** a School of Pharmacy, Tianjin Medical University, Tianjin 300070, People’s Republic of China; b Medical College of Chinese People’s Armed Police Forces, Tianjin 300162, People’s Republic of China; c Tianjin Key Laboratory for Biomarkers of Occupational, and Environmental Hazards, Tianjin 300162, People’s Republic of China

## Abstract

In title compound, C_16_H_17_FN_2_O_2_, the cyclo­hexane ring adopts a chair conformation.. The crystal packing is stabilized by weak π–π stacking inter­actions [centroid–centroid distance = 3.503 (5) Å] and inter­molecular C—H⋯O, N—H⋯O and N—H⋯F hydrogen-bond inter­actions.

## Related literature

For the biological activity of the title compound and its deriv­atives, see: Souli *et al.* (2008 ▶); Chai *et al.* (2006 ▶); Radwan *et al.* (2007 ▶); Karthikeyan *et al.* (2009 ▶). For the preparation, see: Bacher *et al.* (2001 ▶). For bond-length and angle data for similar structures, see: Liu *et al.* (2011 ▶); Sonar *et al.* (2006 ▶).
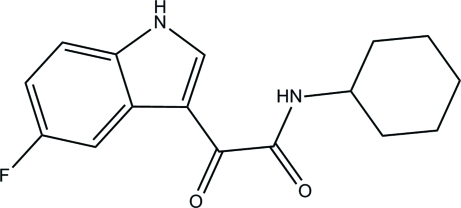


## Experimental

### 

#### Crystal data

C_16_H_17_FN_2_O_2_
*M* *_r_* = 288.32
Monoclinic, 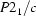

*a* = 11.5065 (15) Å
*b* = 9.7666 (12) Å
*c* = 12.3139 (16) Å
β = 96.639 (5)°
*V* = 1374.5 (3) Å^3^
*Z* = 4
Mo *K*α radiation
μ = 0.10 mm^−1^
*T* = 113 K
0.20 × 0.16 × 0.12 mm

#### Data collection

Rigaku Saturn CCD area-detector diffractometer
Absorption correction: multi-scan (*CrystalClear*; Rigaku, 2005 ▶) *T*_min_ = 0.980, *T*_max_ = 0.988
18432 measured reflections
3717 independent reflections
3007 reflections with *I* > 2σ(*I*)
*R*_int_ = 0.035

#### Refinement

*R*[*F* ^2^ > 2σ(*F* ^2^)] = 0.037
*wR*(*F* ^2^) = 0.098
*S* = 1.04
3717 reflections
198 parameters
H atoms treated by a mixture of independent and constrained refinement
Δρ_max_ = 0.27 e Å^−3^
Δρ_min_ = −0.28 e Å^−3^

### 

Data collection: *CrystalClear* (Rigaku, 2005 ▶); cell refinement: *CrystalClear*; data reduction: *CrystalClear*; program(s) used to solve structure: *SHELXS97* (Sheldrick, 2008 ▶); program(s) used to refine structure: *SHELXL97* (Sheldrick, 2008 ▶); molecular graphics: *SHELXTL* (Sheldrick, 2008 ▶); software used to prepare material for publication: *SHELXTL*.

## Supplementary Material

Crystal structure: contains datablock(s) I, global. DOI: 10.1107/S1600536811024299/hg5056sup1.cif

Structure factors: contains datablock(s) I. DOI: 10.1107/S1600536811024299/hg5056Isup2.hkl

Supplementary material file. DOI: 10.1107/S1600536811024299/hg5056Isup3.cml

Additional supplementary materials:  crystallographic information; 3D view; checkCIF report

## Figures and Tables

**Table 1:** Hydrogen-bond geometry (Å, °)

*D*—H⋯*A*	*D*—H	H⋯*A*	*D*⋯*A*	*D*—H⋯*A*
N2—H2⋯F1^i^	0.905 (13)	2.275 (13)	3.1234 (11)	156.1 (11)
N1—H1⋯O2^ii^	0.939 (14)	1.863 (14)	2.7786 (11)	164.5 (13)
C8—H8⋯O1^iii^	0.95	2.31	3.0586 (12)	136

Symmetry codes: (i) 
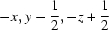
; (ii) 
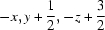
; (iii) 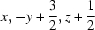.

**Fig. 1.**
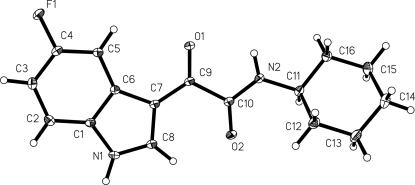
View of the title compound, with displacement ellipsoids drawn at the 40% probability level.

## supplementary crystallographic information

### Comment

Indole and their derivatives are well known as substances with biologically activity such as anti-cancer (Souli *et al.*, 2008), anti-virus(Chai *et al.*, 2006), anti-tubercular (Karthikeyan *et al.*, 2009), and anti-inflammatory (Radwan *et al.*, 2007). In recent years, our recent study is paying attention to synthesize different kinds of indole derivatives with improved bioactivities. In this paper, we reported the crystal structure of title compound.

In title compound, C_16_H_17_FN_2_O_2_, bond lengths and angles are normal and in good agreement with those reported previously (Liu *et al.*, 2011; Sonar, *et al.*, 2006). The cyclohexane ring (C11—C16) adopts a chair conformation. π—π interactions are indicated by the short distance (*Cg*1···*Cg*2 distance of 3.503 (5) Å, symmetry code: -*x*,1 - *y*,-*z*) between the centroids of the pyrrole ring (N1/C1/C6—C8) (*Cg*1) and benzene ring C1—C6 (*Cg*2) (Table 1). There are weaker C—H···O N—H···F and N—H···O intermolecular interactions, which stabilized the structure (Table 1)

### Experimental

The target compound was synthesized by two steps. Oxalyl chloride was added dropwise to 5-fluorine indole in dry ether. 5-fluorine indole-3-yl-glyoxyl chloride which was the crude product, cyclohexane, two drops of triethylamine were in dry dichloromethane. The reaction mixture was washed with water and dried over Na_2_SO_4_ and concentrated *in vacuo* (Bacher *et al.*, 2001). The residue was resolved in a methanol solution. Slow evaporation over two week at room temperature gave light-white crystals suitable for X-ray analysis.

### Refinement

All C H atoms were found on difference maps, with C—H = 0.95–1.00 Å and H atoms bonded N were refined freely with N—H = 0.90 and 0.94 Å and included in the final cycles of refinement using a riding model, with *U*_iso_(H) = 1.2*U*_eq_(C) for aryl and methylene H atoms.

### Crystal data

**Table:**

C_16_H_17_FN_2_O_2_	*F*(000) = 608
*M**_r_* = 288.32	*D*_x_ = 1.393 Mg m^−^^3^
Monoclinic, *P*2_1_/*c*	Mo *K*α radiation, λ = 0.71073 Å
Hall symbol: -P 2ybc	Cell parameters from 5346 reflections
*a* = 11.5065 (15) Å	θ = 1.7–29.1°
*b* = 9.7666 (12) Å	µ = 0.10 mm^−^^1^
*c* = 12.3139 (16) Å	*T* = 113 K
β = 96.639 (5)°	Prism, colorless
*V* = 1374.5 (3) Å^3^	0.20 × 0.16 × 0.12 mm
*Z* = 4	

### Data collection

**Table:**

Rigaku Saturn CCD area-detector diffractometer	3717 independent reflections
Radiation source: rotating anode	3007 reflections with *I* > 2σ(*I*)
multilayer	*R*_int_ = 0.035
Detector resolution: 14.63 pixels mm^-1^	θ_max_ = 29.2°, θ_min_ = 2.7°
ω and φ scans	*h* = −15→15
Absorption correction: multi-scan (*CrystalClear*; Rigaku, 2005)	*k* = −12→13
*T*_min_ = 0.980, *T*_max_ = 0.988	*l* = −16→16
18432 measured reflections	

### Refinement

**Table:**

Refinement on *F*^2^	Primary atom site location: structure-invariant direct methods
Least-squares matrix: full	Secondary atom site location: difference Fourier map
*R*[*F*^2^ > 2σ(*F*^2^)] = 0.037	Hydrogen site location: inferred from neighbouring sites
*wR*(*F*^2^) = 0.098	H atoms treated by a mixture of independent and constrained refinement
*S* = 1.04	*w* = 1/[σ^2^(*F*_o_^2^) + (0.0605*P*)^2^] where *P* = (*F*_o_^2^ + 2*F*_c_^2^)/3
3717 reflections	(Δ/σ)_max_ = 0.001
198 parameters	Δρ_max_ = 0.27 e Å^−^^3^
0 restraints	Δρ_min_ = −0.28 e Å^−^^3^

### Special details

**Table:**

Geometry. All e.s.d.'s (except the e.s.d. in the dihedral angle between two l.s. planes) are estimated using the full covariance matrix. The cell e.s.d.'s are taken into account individually in the estimation of e.s.d.'s in distances, angles and torsion angles; correlations between e.s.d.'s in cell parameters are only used when they are defined by crystal symmetry. An approximate (isotropic) treatment of cell e.s.d.'s is used for estimating e.s.d.'s involving l.s. planes.
Refinement. Refinement of *F*^2^ against ALL reflections. The weighted *R*-factor *wR* and goodness of fit *S* are based on *F*^2^, conventional *R*-factors *R* are based on *F*, with *F* set to zero for negative *F*^2^. The threshold expression of *F*^2^ > σ(*F*^2^) is used only for calculating *R*-factors(gt) *etc*. and is not relevant to the choice of reflections for refinement. *R*-factors based on *F*^2^ are statistically about twice as large as those based on *F*, and *R*- factors based on ALL data will be even larger.

### Fractional atomic coordinates and isotropic or equivalent isotropic displacement parameters (Å^2^)

**Table:**

	*x*	*y*	*z*	*U*_iso_*/*U*_eq_	
F1	−0.25500 (5)	1.05586 (6)	0.22558 (5)	0.02199 (16)	
O1	0.03563 (6)	0.68310 (7)	0.38752 (6)	0.01933 (17)	
O2	0.10442 (6)	0.59789 (7)	0.66261 (6)	0.01922 (17)	
N1	−0.11616 (7)	0.93484 (9)	0.65153 (7)	0.01534 (18)	
N2	0.18874 (7)	0.53328 (8)	0.51269 (7)	0.01509 (18)	
C1	−0.16319 (8)	0.97805 (10)	0.54839 (8)	0.0138 (2)	
C2	−0.24187 (9)	1.08328 (10)	0.51989 (8)	0.0164 (2)	
H2A	−0.2737	1.1364	0.5739	0.020*	
C3	−0.27217 (8)	1.10769 (10)	0.40949 (8)	0.0167 (2)	
H3	−0.3255	1.1788	0.3859	0.020*	
C4	−0.22354 (9)	1.02687 (10)	0.33359 (8)	0.0156 (2)	
C5	−0.14663 (8)	0.92118 (9)	0.35915 (8)	0.0140 (2)	
H5	−0.1162	0.8682	0.3042	0.017*	
C6	−0.11544 (8)	0.89560 (9)	0.47050 (8)	0.01231 (19)	
C7	−0.03768 (8)	0.79970 (10)	0.53188 (7)	0.01268 (19)	
C8	−0.04120 (8)	0.83109 (9)	0.64156 (8)	0.0141 (2)	
H8	0.0030	0.7858	0.7009	0.017*	
C9	0.03360 (8)	0.69899 (10)	0.48630 (7)	0.01308 (19)	
C10	0.11288 (8)	0.60472 (9)	0.56399 (8)	0.0135 (2)	
C11	0.26943 (8)	0.43341 (10)	0.56915 (8)	0.0150 (2)	
H11	0.2231	0.3700	0.6112	0.018*	
C12	0.36197 (9)	0.50230 (11)	0.64966 (9)	0.0204 (2)	
H12A	0.3235	0.5562	0.7034	0.024*	
H12B	0.4092	0.5657	0.6099	0.024*	
C13	0.44143 (10)	0.39422 (12)	0.70913 (9)	0.0249 (3)	
H13A	0.5025	0.4399	0.7597	0.030*	
H13B	0.3948	0.3352	0.7530	0.030*	
C14	0.49948 (9)	0.30599 (11)	0.62842 (9)	0.0229 (2)	
H14A	0.5538	0.3632	0.5912	0.028*	
H14B	0.5457	0.2328	0.6688	0.028*	
C15	0.40945 (9)	0.24164 (10)	0.54345 (9)	0.0205 (2)	
H15A	0.3630	0.1735	0.5795	0.025*	
H15B	0.4503	0.1930	0.4884	0.025*	
C16	0.32700 (8)	0.34877 (10)	0.48594 (8)	0.0168 (2)	
H16A	0.3716	0.4100	0.4418	0.020*	
H16B	0.2659	0.3024	0.4358	0.020*	
H1	−0.1231 (12)	0.9799 (14)	0.7178 (12)	0.043 (4)*	
H2	0.1848 (11)	0.5448 (12)	0.4395 (11)	0.027 (3)*	

### Atomic displacement parameters (Å^2^)

**Table:**

	*U*^11^	*U*^22^	*U*^33^	*U*^12^	*U*^13^	*U*^23^
F1	0.0241 (3)	0.0273 (3)	0.0137 (3)	0.0058 (3)	−0.0016 (2)	0.0056 (2)
O1	0.0251 (4)	0.0224 (4)	0.0100 (3)	0.0067 (3)	0.0000 (3)	−0.0008 (3)
O2	0.0269 (4)	0.0197 (4)	0.0110 (3)	0.0042 (3)	0.0019 (3)	0.0041 (3)
N1	0.0181 (4)	0.0177 (4)	0.0103 (4)	−0.0015 (3)	0.0018 (3)	−0.0032 (3)
N2	0.0171 (4)	0.0157 (4)	0.0121 (4)	0.0022 (3)	−0.0002 (3)	0.0017 (3)
C1	0.0145 (4)	0.0148 (5)	0.0118 (4)	−0.0030 (4)	0.0008 (3)	−0.0013 (3)
C2	0.0154 (5)	0.0157 (5)	0.0187 (5)	−0.0010 (4)	0.0035 (4)	−0.0036 (4)
C3	0.0141 (5)	0.0144 (5)	0.0214 (5)	0.0009 (4)	0.0011 (4)	0.0006 (4)
C4	0.0160 (5)	0.0179 (5)	0.0123 (5)	−0.0020 (4)	−0.0013 (3)	0.0023 (4)
C5	0.0153 (5)	0.0148 (5)	0.0119 (5)	−0.0015 (4)	0.0013 (3)	−0.0011 (3)
C6	0.0129 (4)	0.0124 (4)	0.0116 (4)	−0.0022 (4)	0.0014 (3)	−0.0004 (3)
C7	0.0143 (4)	0.0132 (4)	0.0102 (4)	−0.0028 (4)	0.0002 (3)	0.0004 (3)
C8	0.0154 (4)	0.0150 (5)	0.0116 (5)	−0.0027 (4)	0.0006 (3)	−0.0001 (3)
C9	0.0149 (4)	0.0136 (4)	0.0102 (4)	−0.0024 (4)	−0.0006 (3)	0.0005 (3)
C10	0.0162 (5)	0.0116 (4)	0.0122 (4)	−0.0025 (4)	0.0000 (3)	0.0007 (3)
C11	0.0148 (5)	0.0143 (5)	0.0154 (5)	0.0006 (4)	−0.0010 (4)	0.0022 (4)
C12	0.0193 (5)	0.0202 (5)	0.0200 (5)	0.0004 (4)	−0.0041 (4)	−0.0017 (4)
C13	0.0211 (5)	0.0283 (6)	0.0230 (6)	0.0024 (5)	−0.0071 (4)	0.0016 (4)
C14	0.0163 (5)	0.0217 (5)	0.0295 (6)	0.0023 (4)	−0.0027 (4)	0.0053 (4)
C15	0.0173 (5)	0.0164 (5)	0.0277 (6)	0.0010 (4)	0.0020 (4)	0.0012 (4)
C16	0.0154 (5)	0.0162 (5)	0.0185 (5)	−0.0009 (4)	0.0007 (4)	0.0003 (4)

### Geometric parameters (Å, °)

**Table:**

F1—C4	1.3671 (11)	C7—C9	1.4358 (14)
O1—C9	1.2291 (11)	C8—H8	0.9500
O2—C10	1.2313 (12)	C9—C10	1.5470 (13)
N1—C8	1.3454 (13)	C11—C12	1.5249 (14)
N1—C1	1.3874 (12)	C11—C16	1.5261 (14)
N1—H1	0.939 (14)	C11—H11	1.0000
N2—C10	1.3324 (13)	C12—C13	1.5270 (14)
N2—C11	1.4655 (12)	C12—H12A	0.9900
N2—H2	0.905 (13)	C12—H12B	0.9900
C1—C2	1.3877 (14)	C13—C14	1.5265 (16)
C1—C6	1.4113 (13)	C13—H13A	0.9900
C2—C3	1.3846 (14)	C13—H13B	0.9900
C2—H2A	0.9500	C14—C15	1.5206 (14)
C3—C4	1.3895 (14)	C14—H14A	0.9900
C3—H3	0.9500	C14—H14B	0.9900
C4—C5	1.3723 (14)	C15—C16	1.5300 (13)
C5—C6	1.3992 (13)	C15—H15A	0.9900
C5—H5	0.9500	C15—H15B	0.9900
C6—C7	1.4466 (13)	C16—H16A	0.9900
C7—C8	1.3901 (13)	C16—H16B	0.9900
			
C8—N1—C1	109.38 (8)	N2—C11—C12	111.79 (8)
C8—N1—H1	123.5 (9)	N2—C11—C16	110.02 (8)
C1—N1—H1	126.2 (9)	C12—C11—C16	110.54 (8)
C10—N2—C11	122.51 (8)	N2—C11—H11	108.1
C10—N2—H2	116.5 (8)	C12—C11—H11	108.1
C11—N2—H2	120.8 (8)	C16—C11—H11	108.1
N1—C1—C2	129.11 (9)	C11—C12—C13	109.95 (8)
N1—C1—C6	107.90 (9)	C11—C12—H12A	109.7
C2—C1—C6	122.99 (9)	C13—C12—H12A	109.7
C3—C2—C1	117.30 (9)	C11—C12—H12B	109.7
C3—C2—H2A	121.3	C13—C12—H12B	109.7
C1—C2—H2A	121.3	H12A—C12—H12B	108.2
C2—C3—C4	119.17 (9)	C14—C13—C12	111.15 (9)
C2—C3—H3	120.4	C14—C13—H13A	109.4
C4—C3—H3	120.4	C12—C13—H13A	109.4
F1—C4—C5	118.04 (9)	C14—C13—H13B	109.4
F1—C4—C3	117.05 (9)	C12—C13—H13B	109.4
C5—C4—C3	124.91 (9)	H13A—C13—H13B	108.0
C4—C5—C6	116.40 (9)	C15—C14—C13	111.51 (8)
C4—C5—H5	121.8	C15—C14—H14A	109.3
C6—C5—H5	121.8	C13—C14—H14A	109.3
C5—C6—C1	119.23 (9)	C15—C14—H14B	109.3
C5—C6—C7	134.48 (9)	C13—C14—H14B	109.3
C1—C6—C7	106.27 (8)	H14A—C14—H14B	108.0
C8—C7—C9	127.81 (9)	C14—C15—C16	111.87 (8)
C8—C7—C6	106.20 (8)	C14—C15—H15A	109.2
C9—C7—C6	125.88 (8)	C16—C15—H15A	109.2
N1—C8—C7	110.23 (8)	C14—C15—H15B	109.2
N1—C8—H8	124.9	C16—C15—H15B	109.2
C7—C8—H8	124.9	H15A—C15—H15B	107.9
O1—C9—C7	123.40 (9)	C11—C16—C15	110.71 (8)
O1—C9—C10	117.35 (8)	C11—C16—H16A	109.5
C7—C9—C10	119.25 (8)	C15—C16—H16A	109.5
O2—C10—N2	124.75 (9)	C11—C16—H16B	109.5
O2—C10—C9	122.28 (8)	C15—C16—H16B	109.5
N2—C10—C9	112.97 (8)	H16A—C16—H16B	108.1
			
C8—N1—C1—C2	−178.59 (10)	C6—C7—C8—N1	1.51 (10)
C8—N1—C1—C6	0.56 (11)	C8—C7—C9—O1	−175.91 (9)
N1—C1—C2—C3	178.08 (9)	C6—C7—C9—O1	−0.28 (16)
C6—C1—C2—C3	−0.96 (14)	C8—C7—C9—C10	3.92 (15)
C1—C2—C3—C4	0.18 (14)	C6—C7—C9—C10	179.56 (8)
C2—C3—C4—F1	−179.24 (8)	C11—N2—C10—O2	2.07 (15)
C2—C3—C4—C5	0.59 (15)	C11—N2—C10—C9	−177.56 (8)
F1—C4—C5—C6	179.28 (8)	O1—C9—C10—O2	−168.77 (9)
C3—C4—C5—C6	−0.55 (15)	C7—C9—C10—O2	11.39 (14)
C4—C5—C6—C1	−0.23 (13)	O1—C9—C10—N2	10.87 (12)
C4—C5—C6—C7	−178.32 (10)	C7—C9—C10—N2	−168.98 (9)
N1—C1—C6—C5	−178.22 (8)	C10—N2—C11—C12	−68.08 (12)
C2—C1—C6—C5	1.00 (14)	C10—N2—C11—C16	168.70 (8)
N1—C1—C6—C7	0.37 (10)	N2—C11—C12—C13	178.16 (8)
C2—C1—C6—C7	179.58 (9)	C16—C11—C12—C13	−58.92 (11)
C5—C6—C7—C8	177.15 (10)	C11—C12—C13—C14	57.56 (12)
C1—C6—C7—C8	−1.12 (10)	C12—C13—C14—C15	−54.88 (12)
C5—C6—C7—C9	0.74 (17)	C13—C14—C15—C16	53.30 (12)
C1—C6—C7—C9	−177.53 (9)	N2—C11—C16—C15	−178.68 (8)
C1—N1—C8—C7	−1.32 (11)	C12—C11—C16—C15	57.37 (11)
C9—C7—C8—N1	177.83 (9)	C14—C15—C16—C11	−54.54 (11)

### Hydrogen-bond geometry (Å, °)

**Table:**

*D*—H···*A*	*D*—H	H···*A*	*D*···*A*	*D*—H···*A*
N2—H2···F1^i^	0.905 (13)	2.275 (13)	3.1234 (11)	156.1 (11)
N1—H1···O2^ii^	0.939 (14)	1.863 (14)	2.7786 (11)	164.5 (13)
C8—H8···O1^iii^	0.95	2.31	3.0586 (12)	136.

Symmetry codes: (i) −*x*, *y*−1/2, −*z*+1/2; (ii) −*x*, *y*+1/2, −*z*+3/2; (iii) *x*, −*y*+3/2, *z*+1/2.
